# Potential Mechanisms of Mindfulness in Improving Sleep and Distress

**DOI:** 10.1007/s12671-017-0796-9

**Published:** 2017-08-29

**Authors:** Way K. W. Lau, Mei-Kei Leung, Yun-Kwok Wing, Tatia M. C. Lee

**Affiliations:** 1 Laboratory of Neuropsychology, The Jockey Club Tower, The University of Hong Kong, Room 656, Pokfulam Road, Hong Kong, China; 2 Department of Psychiatry, Faculty of Medicine, The Chinese University of Hong Kong, Hong Kong, China; 3 The State Key Laboratory of Brain and Cognitive Sciences, The University of Hong Kong, Hong Kong, China

**Keywords:** Mindfulness, Sleep, Psychological distress, Mediation, Acceptance

## Abstract

The mechanisms of mindfulness-improved sleep quality are not extensively studied. Recently, attention monitoring/awareness and acceptance in mindfulness have been proposed to be the underlying mechanisms that tackle distress and related disorders. The current study tested if acceptance moderated the relationship of awareness with psychological distress and sleep quality, and verified that psychological distress mediated the relationship between mindfulness and sleep quality in a group of community-dwelling healthy adults. Three hundred and sixty-four healthy Chinese non-meditators (age 18–65, 59% female) completed a set of online self-reported questionnaires in Chinese via SurveyMonkey. Awareness and acceptance were measured by *Observe* and *Nonreact* facets in the Five Facet Mindfulness Questionnaire (FFMQ), respectively. General psychological distress levels and sleep quality were reflected in the global score of the Depression Anxiety and Stress Scales (DASS) and the Pittsburgh Sleep Quality Index (PSQI), respectively. Model 1 and model 8 in the PROCESS macro for SPSS were used to assess the moderation and moderated mediation effects. Increased level of acceptance (*Nonreact*) weakened the positive relationship between awareness (*Observe*) and poor sleep quality (*β* = −0.0154, *p* = 0.0123), which was partially mediated through perceived psychological distress (*β* = −0.0065, 95% bias-corrected bootstrap CI = −0.0128, −0.0004) in a group of community-dwelling healthy adults. Our findings suggested that awareness and acceptance could be the mechanisms of mindfulness interventions in improving sleep quality, partly via reducing psychological stress.

**Electronic supplementary material:**

The online version of this article (doi:10.1007/s12671-017-0796-9) contains supplementary material, which is available to authorized users.

## Introduction

Sleep difficulty or poor sleep quality is commonly reported worldwide. The prevalence of self-reported insomnia in western countries ranges from 10 to 48% (Ohayon and Paiva 2005; Ohayon and Smirne 2002); the prevalence of insomnia among Chinese adults in Hong Kong ranges from 12 to 40% (Zhang et al. 2009; Wong and Fielding 2011). Sleep difficulty or poor sleep quality is highly associated with psychological distress. For instance, people with untreated insomnia had a higher prevalence of having major depression or anxiety (odds ratio = 39.8), compared to those without insomnia in a large community sample (*N* = 7954, Ford and Kamerow 1989). In agreement with the previous report, sleep disturbances and subjective sleep quality were found to be strongly associated with depression in a large group of postpartum women (*N* = 4191, Dorheim et al. 2009). Reciprocally, high perceived stress was a significant predictor of poor sleep quality in a group of medical students (*N* = 305, Alsaggaf et al. 2016). On the other hand, psychological distress including anxiety and depression, and increased arousal, a key factor of chronic insomnia, were found to be significant predictors of the maintenance of insomnia in a large group of random samples (*N* = 1936, age range = 20–60 years old) from four counties in Sweden (Jansson and Linton 2007). These findings indicate the strong association between poor sleep quality and psychological distress.

Ong, Ulmer, and Manber (2012) presented a conceptual framework for the cognitive mechanisms of insomnia, emphasizing that mindfulness/awareness and acceptance are essential components in treating insomnia. They proposed that insomnia was caused by increased arousal, in which two levels of sleep-related cognitive arousal were presented in the context of insomnia. Primary arousal consists of cognitive activity, such as thoughts that directly impair sleep. Secondary or metacognitive arousal is related to the awareness and interpretation of primary arousal, which includes how positively/negatively one evaluates his/her thoughts and beliefs about sleep. Mindfulness interventions specifically target the secondary arousal to increase awareness of the mental and physical states when experiencing insomnia symptoms and then to shift mental processes to an adaptive stance (acceptance) in response to these symptoms (see Ong et al. 2012 for review). In a recent study, long-lasting emotional distress induced by an introduction to examples of shameful experiences was found to be a significant mediator of the positive association between thought-like nocturnal mentation (predictor) and hyperarousal (dependent variable) in 1199 middle-aged participants (Wassing et al. 2016). It is possible that mindfulness interventions by reducing psychological distress and hence arousal can improve sleep quality.

Mindfulness is defined as the awareness that emerges through paying attention on purpose, in the present moment, and nonjudgmentally to the unfolding of experience moment by moment (Kabat-Zinn 2003). In the past decade, extensive studies provided evidence supporting that mindfulness was associated with a wide variety of beneficial psychological outcomes, such as emotion and attention regulation (Brown et al. 2007), reduction of stress, anxiety, and depression (see Khoury et al. 2013 for review). In addition, mindfulness training was reported to be effective for insomnia (Gong et al. 2016) and improving sleep quality (Black et al. 2015). The beneficial effects of mindfulness on sleep problems have been proven in different populations. For instance, higher levels of dispositional mindfulness were associated with better quality of sleep and better physical health in female college students (Murphy et al. 2012). On the other hand, mindfulness interventions were shown to improve sleep quality in cancer patients (Rouleau et al. 2015), healthy young adults (van der Riet et al. 2015), healthy elderly people (Black et al. 2015), and elderly subjects with chronic insomnia (Zhang et al. 2015). A recent meta-analysis demonstrated that 6 weeks to 2 months of mindfulness trainings significantly improved sleep quality in people with chronic insomnia or other sleep problems (Gong et al. 2016). However, the mechanisms of mindfulness that improved sleep problems were not extensively studied.

To understand why mindfulness training could be beneficial to sleep problems, Lindsay and Creswell (2016) proposed the Monitor and Acceptance Theory (MAT) to explain the potential underlying mechanisms of mindfulness in alleviating stress and its related disorders. They proposed that attention monitoring/awareness was the key for improving cognitive outcomes and enhancing attention to both positive and negative affective information, whereas both attention monitoring/awareness and acceptance were essential for improving affective regulation (Lindsay and Creswell 2016). This theory was supported by a few studies that investigated the modulating effect of acceptance on the relationship between awareness and psychological distress outcomes. For instance, a high tendency to monitor [measured by *Observe* facet in the Five Facet Mindfulness Questionnaire (FFMQ), Baer et al. 2006] was associated with more depressive symptoms among students with low levels of acceptance (measured by *Nonreact* facet in the FFMQ), whereas a high tendency to monitor was associated with less depressive symptoms among students with high levels of acceptance (Barnes and Lynn 2010). Many of these studies, however, examined the interacting effects between awareness and acceptance on students, which limits the generalizability of the findings. Given psychological distress was one of the significant predictors of the maintenance of insomnia (Jansson and Linton 2007), cultivating both awareness and acceptance could possibly reduce sleep problems via alleviating psychological distress.

Taken together, the literature suggests that awareness and acceptance may be the underlying mechanisms of mindfulness in reducing psychological distress and improving sleep quality. It is likely that psychological distress could mediate the relationship between mindfulness and sleep quality. The aims of this study were to test the moderating effect of acceptance on the relationship of awareness with psychological distress and sleep quality, and to verify whether psychological distress mediated the relationship of the interaction between awareness and acceptance with sleep quality in a group of community dwelling healthy adults. We hypothesized that both high levels of awareness and acceptance were essential for improving sleep quality, which was at least partially mediated by reducing psychological distress.

## Method

### Participants

Three hundred and seventy-one out of 438 subjects completed all parts of the questionnaires (completion rate = 84.70%). Influential multivariate outliers identified by Mahalanobis’ distances were removed (*N* = 6). One subject with meditation background was also excluded, yielding a final set of 364 samples (214 female, age range = 18–65, Mean_Age_ = 37.75, SD_Age_ = 9.55) for analyses. The demographic data for the 364 subjects are reported in Table 1. The average global FFMQ score that represents a general trait of mindfulness in our subjects was 125.43 (SD = 14.16). The internal consistency of the global FFMQ score and the subscales was acceptable to good (Cronbach alpha = 0.74–0.90, see Table 2). For psychological distress, our subjects were regarded as normal in all three aspects in the DASS [average stress scores 5.65 (SD = 4.02); average anxiety scores 3.16 (SD = 3.00); average depression scores 3.56 (SD = 3.44)]. The internal consistency of the global DASS score and the three subscales was acceptable to good (Cronbach alpha = 0.77–0.92, see Table 2). In average, our subjects demonstrated some degrees of sleep problem (69% of subjects with global PSQI score > 5). The average global PSQI score was 7.17 (SD = 2.95). The internal consistency of the global PSQI score obtained in our samples was marginally acceptable (Cronbach alpha = 0.67, see Table 2).

**Table 1:** Demographics

Variables	Mean (SD) / *N* (%)
Age (years old): mean (SD) (*N* = 364)	37.75 (9.55)
- 18–25 (years old): *N* (%)	30 (8.24)
- 26–45 (years old): *N* (%)	253 (69.51)
- 46–65 (years old): *N* (%)	81 (22.25)
Gender (female): *N* (%) (*N* = 364)	214 (58.79)
Education (years): Mean (SD) (*N* = 364)	17.55 (2.80)
BMI: mean (SD) (*N* = 362)	22.03 (7.08)
Marriage: *N* (%) (*N* = 364)	
- Single	215 (59.07)
- Married	133 (36.54)
- Divorced	12 (3.30)
- Widowed	4 (1.10)
Income (HKD): *N* (%) (*N* = 364)	
- < $5000	15 (4.12)
- $5000–$10,000	8 (2.20)
- $10,001–$20,000	53 (14.56)
- $20,001–$30,000	55 (15.11)
- $30,001–$40,000	63 (17.31)
- $ > 40,000	143 (39.29)
- Not reported	27 (7.42)

*N* number of subject, *SD* standard derivation; *HKD* = Hong Kong dollar

**Table 2:** Descriptive statistics and internal consistency of the administrated psychological constructs and their correlation (*N = 364*)

	Mean (SD)	Range	*α*	Pearson’s correlation coefficient									
				2.	3.	4.	5.	6.	7.	8.	9.	10.	11.
1. FFMQ	125.43 (14.16)	84–184	0.87	.513***	.739***	.687***	.379***	.603***	−.534***	−.516***	−.443***	−.481***	−.326***
2. *Observe* (8-items)	23.54 (5.25)	11–39	0.81		.272***	.055	−.234***	.402***	−.013	−.008	.041	−.040	−.015
3. *Describe* (8-items)	27.51 (5.45)	12–40	0.90			.371***	.088	.368***	−.350***	−.315***	−.330***	−.320***	−.225***
4. *Act with Awareness* (8-items)	27.68 (4.66)	14–40	0.86				.366***	.232***	−.516***	−.488***	−.434***	−.479***	−.360***
5. *Nonjudge* (8-items)	25.06 (4.72)	10–39	0.81					−.056	−.423***	−.403***	−.364***	−.365***	−.196***
6. *Nonreact* (7-items)	21.64 (3.70)	10–32	0.74						−.279***	−.314***	−.246***	−.202***	−.187***
7. DASS	12.37 (9.31)	0–46	0.92							.930***	.834***	.863***	.452***
8. *Stress* (7-items)	5.65 (4.02)	0–19	0.84								.711***	.704***	.410***
9. *Anxiety* (7-items)	3.16 (3.00)	0–17	0.77									.607***	.466***
10. *Depression* (7-items)	3.56 (3.44)	0–18	0.84										.342***
11. PSQI (7 components)	7.17 (2.95)	1–17	0.67										

*α* Cronbach alpha, *DASS* Depression Anxiety and Stress Scales, *FFMQ* Five Facet Mindfulness Questionnaire, *PSQI* Pittsburgh Sleep Quality Index. Significant Pearson’s correlation coefficients were asterisked (***p < 0.001, two-tailed)

### Procedure

This was a cross-sectional study. A convenience sampling method was adopted. Healthy adults were recruited through email or online platforms. Participants completed the consent form and a set of online self-reported questionnaires in Chinese via SurveyMonkey (SurveyMonkey Inc., Palo Alto, California, USA). Participants with meditation backgrounds and/or who failed to complete the whole set of questionnaires were excluded from the analysis. About 20% of the subjects who completed the whole set of questionnaires would receive 100 Hong Kong dollars through a lucky draw, as incentives for the appreciation of their contribution of time and participation. The winners could also authorize us to help them donate the money to charities. This study was approved by the Research Ethics Committee of the University of Hong Kong.

In addition to the tested hypotheses in the current study, we originally intended to test the mediating role of sleep problems in the relationship between mindfulness, mental health, quality of life, and cognition. Having considered the inclusion of these additional measures would diverse the focus of this study, therefore, that part was not included in the current study.

### Measures

#### Five Facet Mindfulness Questionnaire

The FFMQ measures five facets of mindfulness: *Observe* (8 items; e.g., “When I’m walking, I deliberately notice the sensations of my body moving”), *Describe* (8 items; e.g., “I’m good at finding words to describe my feelings”), *Act with awareness* (8 items; e.g., “When I do things, my mind wanders off and I’m easily distracted”), *Nonjudge* (8 items; e.g., “I criticize myself for having irrational or inappropriate emotions”), and *Nonreact* (7 items; e.g., “I perceive my feelings and emotions without having to react to them”). It has 39 items and is rated on a 5-point Likert scale, ranging from 1 (never or very rarely true) to 5 (very often or always true) (Baer et al. 2006). The *Observe* and *Nonreact* facets were extracted to reflect the awareness and acceptance, respectively. Although both the *Nonjudge* and *Nonreact* facets were regarded as useful measures for acceptance (Baer et al. 2006), only the *Nonreact* facet interacted with the *Observe* facet to influence depressive symptoms (Barnes and Lynn 2010; Desrosiers et al. 2014). Therefore, the *Observe* and *Nonreact* facets were chosen as a representative of awareness and acceptance, respectively, in this study.

#### Pittsburgh Sleep Quality Index

The Chinese version of PSQI assesses seven dimensions of sleep quality over the past month: subjective sleep quality, sleep latency, sleep duration, habitual sleep efficiency, sleep disturbances, use of sleep medication, and daytime dysfunction (Tsai et al. 2005). A global PSQI score larger than 5 is regarded as having sleep problems. Higher scores indicate more difficulties in sleep. The global PSQI score that reflects general sleep difficulties was used as the outcome variable in the current study.

#### Depression Anxiety and Stress Scales

The DASS was developed by Antony, Bieling, Cox, Enns, and Swinson (1998) to measure the affective states of depression, anxiety, and stress. It consists of 21 items. Each item is rated on a 4-point scale, ranging from 0 (did not apply to me at all) to 3 (applied to me very much). There are seven items on each of the three DASS subscale. The sum of scores from each subscale ranges from 0 to 21, with the higher scores indicating higher severity in that particular aspect. The global DASS score that reflects a general psychological distress (Henry and Crawford 2005) was used as the outcome measure in studying the interaction effect between awareness and acceptance on distress and as the mediator in studying the relationship between mindfulness and sleep quality.

### Data Analyses

Statistical analyses were conducted using the Statistical Package for Social Science (SPSS v22). A *p* value < 0.05 was considered statistically significant.

Normality of the data was tested by Shapiro-Wilk analysis. The global DASS and PSQI scores, as well as the scores from *Observe* and *Nonreact* facets in FFMQ, demonstrated non-normal distributions. These variables were transformed using a two-step transformation method (Templeton 2011), prior to parametric analyses such as Pearson’s correlation and independent *t* test. Briefly, the variable was first transformed into a percentile rank followed by the application of an inverse normal transformation function to the ranked data to form a variable consisting of normally distributed *z*-scores. Enhanced normality using the two-step transformation method was reported to improve the normality of residuals in linear regression modeling (Templeton 2011). Therefore, transformed scores were used in the multiple linear regression models in this study.

The associations among sleep quality, psychological distress, and mindfulness were investigated using Pearson’s correlation analysis (two-tailed, Table 1). In our samples, a significant negative association between age and global DASS score (*r* = −0.246, *p* < 0.001, Pearson’s correlation, two-tailed) and a significant positive association between age and *Nonreact* (*r* = 0.270, *p* < 0.001, Pearson’s correlation, two-tailed) were observed. A small and statistically significant gender difference in *Nonreact* [22.146 ± 3.448 vs 21.338 ± 3.788, male vs female, *t*(362) = 2.075, *p* = 0.039] was also observed. There were no significant associations between BMI and the other measures (*p* > 0.05, Pearson’s correlation, two-tailed), although poor sleep quality was reported to associate with obesity (Araghi et al. 2013). Therefore, only the effects of age and gender were controlled in the multiple linear regression models.

**Fig. 1:**
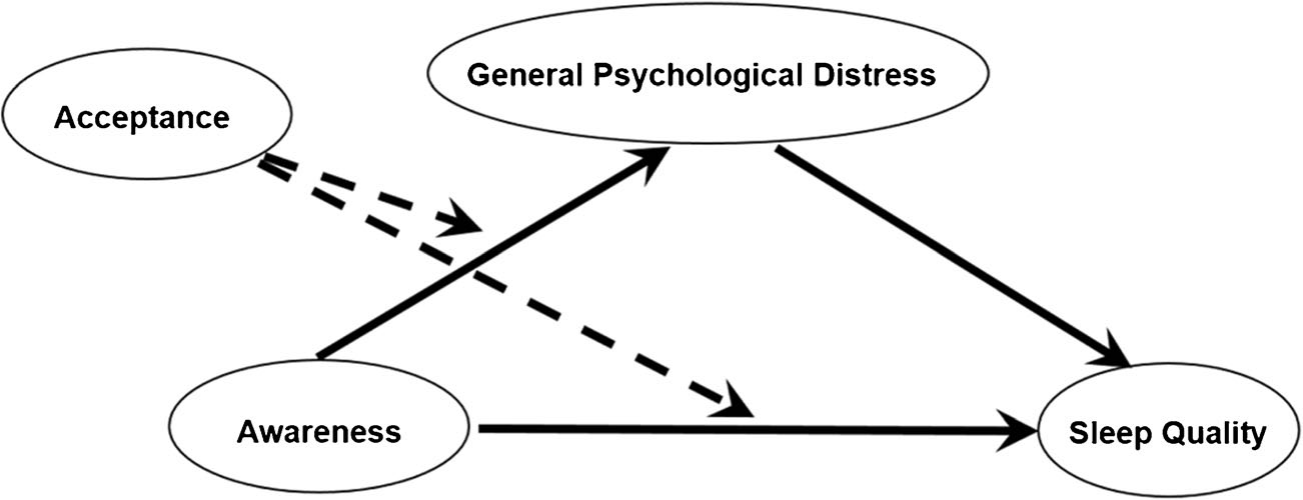
A schematic diagram of the hypothesized moderated mediation pathways. Awareness was measured by *Observe* facet in the self-reported Five Facet Mindfulness Questionnaire (FFMQ); acceptance was measured by *Nonreact* facet in the FFMQ. General psychological distress was reflected in the global score of the Depression Anxiety and Stress Scales (DASS); sleep quality was reflected in the global score of the Pittsburgh Sleep Quality Index (PSQI). The main effect of acceptance is omitted in the diagram for simplicity. Dotted lines represent moderating effects

Moderation and moderated mediation analyses were performed using model 1 and 8, respectively, in the PROCESS macro for SPSS developed by Hayes (2013). Briefly, the scores of *Observe* (predictor) and *Nonreact* (moderator) were mean-centered. The mean-centered values together with their interaction terms were entered in the linear regression model to predict the global DASS score (outcome in model 1; mediator in model 8), and global PSQI score (outcome in model 8, Fig. 1). The effects of covariates (age and gender) were adjusted. The PROCESS macro is based on ordinary least squares regression and adopts a nonparametric bootstrapping procedure (5000 bootstrapped samples in this study), which gives rise to a bias-corrected confidence interval (CI) for effect size inference (Shrout and Bolger 2002). The presence of a significant effect is denoted if zero is not included by the upper and lower bound of 95% CI (Preacher and Hayes 2008).

## Results

**Fig. 2:**
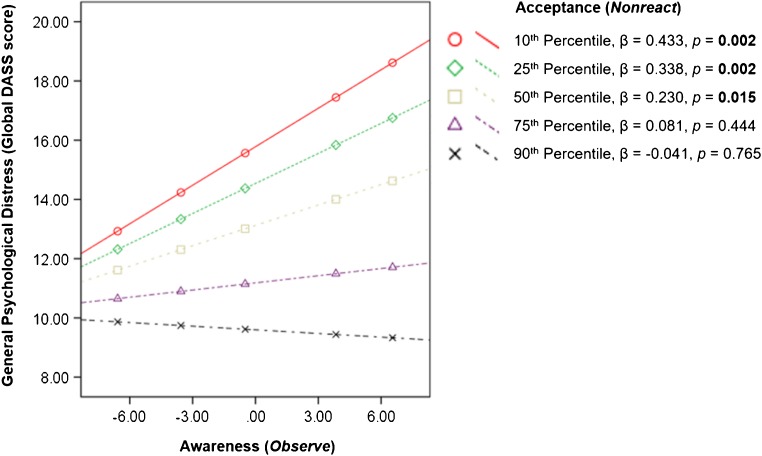
Conditional effects of acceptance on the relationship between awareness and psychological distress. β = Unstandardized coefficient. The effects of age and gender were controlled in the regression model. A *p*-value <0.05 was regarded as significant

The regression model that included *Observe*, *Nonreact*, interaction between *Observe* and *Nonreact*, age, and gender in predicting global DASS score was significant (*F* = 11.049, *R* ^2^ = 0.134, *p* < 0.001). Significant main effects of awareness [*Observe*, unstandardized coefficient (*β*) = 0.204, standard error (SE) = 0.093, *p* = 0.029], and acceptance (*Nonreact*, *β* = −0.628, SE = 0.140, *p* < 0.0001), as well as the interaction between awareness and acceptance (*β* = −0.048, SE = 0.020, *p* = 0.018) on general psychological distress were observed (Table 3). Notably, the positive association between awareness (*Observe*) and general psychological distress was reduced with an increased level of acceptance (*Nonreact*). The positive association between awareness (*Observe*) and general psychological distress became non-significant when the level of acceptance was above the 50th percentile in our samples (Fig. 2).

**Table 3:** Moderating effects of acceptance on the relationship between awareness and general psychological distress *(N = 364)*

Variables	*β*	SE	*p* value	95% CI	
				Lower bound	Upper bound
*Nonreact*	−.628	.140	< .0001	−.904	−.352
*Observe*	.204	.093	.0294	.021	.387
Interaction	−.048	.020	.0181	−.088	−.008
Age	−.177	.049	.0003	−.273	−.081
Gender	.020	.921	.9830	−1.792	1.831

The interaction term was generated by multiplying the mean-centered values of *Nonreact* and *Observe*. The effects of age and gender were controlled

β unstandardized coefficient, *CI* confidence interval, *SE* standard error

The regression model that included *Observe*, *Nonreact*, interaction between *Observe* and *Nonreact*, global DASS score, age and gender in predicting global PSQI score was significant (*F* = 17.302, *R* ^2^ = 0.225, *p* < 0.0001). General psychological distress (*β* = 0.1342, SE = 0.0158, *p* < 0.001), but not awareness (*Observe*) nor acceptance (*Nonreact*), had a main effect on the overall sleep quality (global PSQI score). The interaction effect between awareness (*Observe*) and acceptance (*Nonreact*) on the overall sleep quality was significant (*β* = −0.0154, SE = 0.0061, *p* = 0.0123), after controlling for the effects of age and gender in a linear regression model.

The direct effect of awareness (*Observe*) on the overall sleep quality depended on the level of acceptance (*Nonreact*). The significant positive association between awareness (*Observe*) and poor sleep quality in general was observed only when acceptance (*Nonreact*) level was low (Table 4).

**Table 4:** Conditional direct and indirect effects of awareness on the overall sleep quality (*N = 364*)

Acceptance level		Conditional direct effect				Conditional indirect effect			
%	Mean centered			95% CI				95% CI^a^	
		β	SE	LB	UB	β	SE	LB	UB
10th	− 4.762	.086	.041	.005	.168	.058	.024	.014	.106
25th	− 2.787	.056	.034	− .010	.122	.045	.019	.010	.083
50th	− .532	.021	.029	− .035	.077	.031	.015	.003	.061
75th	2.561	− .027	.032	− .089	.036	.011	.014	− .017	.038
90th	5.087	− .065	.041	− .146	.015	− .006	.017	− .041	.028

^a^Bias-corrected bootstrap confidence intervals. The effects of age and gender were controlled

*β* unstandardized coefficient, *CI* confidence interval, *SE* standard error, *LB* lower bound; *UB* upper bound

The conditional indirect effect of awareness (*Observe*) on the overall sleep quality through general psychological distress was significant (*β* = − 0.0065, SE = 0.0031, 95% bias-corrected bootstrap CI = − 0.0128, − 0.0004). Notably, the positive indirect effect of awareness (*Observe*) on poor sleep quality decreased with an increased level of acceptance (*Nonreact*), and the indirect effect became non-significant when acceptance (*Nonreact*) level was equal to or higher than the 75th percentile (Table 4).

## Discussion

Our findings showed a significant moderating effect of acceptance (*Nonreact*) on the relationship between awareness (*Observe*) and the overall quality of sleep (global PSQI score), which was partially mediated through perceived psychological distress (global DASS score) in a group of community-dwelling healthy adults. These findings indicate that awareness and acceptance, via the reduction of psychological distress, are the likely mechanisms of mindfulness that underpin improvement of sleep quality.

A previous study investigated the moderating role of acceptance, measured by the *Nonreact* facet in the FFMQ, in the relationship between attention monitoring/awareness, measured by the *Observe* facet in FFMQ, and psychological distress on adult samples with mood disorders (Desrosiers et al. 2014). Our findings further confirmed that awareness itself was positively associated with perceived psychological distress, while this positive association was weakened by an increased acceptance (*Nonreact*) level, in a group of community-dwelling healthy adults, providing a better generalizability to the general community compared to the previous report that focused mainly on patients. In addition, we found that acceptance (*Nonreact or Nonjudge*) alone had a strong negative effect on psychological distress, which was consistent with the previous reports from the literature (Brown et al. 2015; Cash and Whittingham 2010).

Specifically, the interaction effect on psychological distress was observed only between the *Observe* and *Nonreact*, but not between the *Observe* and *Nonjudge* facets in our samples (Supplementary Table 1). Despite both the *Nonreact* and *Nonjudge* facets were regarded as useful measures for acceptance, they were distinct from each other in the FFMQ (Baer et al. 2006). For instance, items included in the *Nonreact* facet capture how frequent one is aware of his/her emotions or problems without attaching to them, which reflects acceptance directly, whereas items included in the *Nonjudge* facet ask how frequent one judges his/her emotions or thoughts, and then reverse scoring is used to reflect acceptance. Consistently, a positive correlation between *Observe* and *Nonreact* was commonly reported in non-meditators, whereas a negative correlation between *Observe* and *Nonjudge* was found (Barnes and Lynn 2010; Hamill et al. 2015), indicating a potential difference in the interaction of the *Observe* facet with the *Nonreact* and *Nonjudge* facets. One possible explanation for such a difference is that associations of the *Observe* facet with other mindfulness facets are very sensitive to changes in meditation experience (Baer et al. 2006). We observed significant interaction between the *Observe* and *Nonreact*, which could be unique to non-meditators. Such differential interaction effects of the *Observe* with *Nonreact* and *Nonjudge* on psychological distress and sleep quality may disappear in experienced meditators. More studies are warranted to investigate the differences between the *Nonreact* and *Nonjudge* facets in representing acceptance, and their differential interactions with the *Observe* facet.

The interaction effect between the *Observe* and *Nonreact*, but not the main effect of the *Observe* nor *Nonreact*, on the overall sleep quality was significant. This suggests that both awareness and acceptance are core elements for mindfulness intervention to tackle sleep problems. Notably, a significant positive association between awareness (*Observe*) and poor sleep quality was observed when acceptance (*Nonreact*) level was low. This finding suggests that high levels of attention monitoring/awareness (*Observe*) may exacerbate sleep problems in people with a low level of dispositional acceptance (*Nonreact*) due to the increased perception of and/or attention to maladaptive beliefs and attitudes about sleep (Morin et al. 1993). It is noteworthy that we did not observe any interaction effect between the *Observe* and *Nonjudge* facets on sleep quality (Supplementary Table 2), indicating once again their differential interactions with awareness (*Observe*). Such claim requires more empirical studies to confirm.

Mindfulness interventions cultivate both awareness and acceptance that impacts positively on sleep quality. A recent meta-analysis reported a significant positive effect of mindfulness meditation on sleep quality based on the results of six randomized controlled trials on people with insomnia (Gong et al. 2016). In our samples, we found that the positive association between awareness and poor sleep quality was weakened and became non-significant with an increased acceptance level. Importantly, the positive association between awareness and poor sleep quality was partially mediated through perceived psychological distress and was dependent on the level of acceptance. At low levels of acceptance (≤ 50th percentile), the positive association between attention monitoring/awareness and sleep difficulties was mediated through psychological distress. This phenomenon may be explained by an inability to move away attention from negative thoughts, i.e., rumination, resulting in increased psychological distress and hence, sleep difficulties. The mediating effect was, however, not significant in subjects with high dispositional acceptance levels (> 50th percentile). These findings suggest that acceptance (*Nonreact*) may dissociate the adverse effects among awareness, psychological distress, and poor sleep quality. It is noteworthy that even at high levels of acceptance (i.e., at 90th percentile of *Nonreact*), high levels of awareness (*Observe*) appeared to be unrelated to less psychological distress or better sleep quality, whereas the overall mindfulness (FFMQ total) was negatively correlated to psychological distress and sleep problems. It may be explained by the fact that the levels of *Nonreact* are often low in non-meditators and are usually the lowest among the five facets in the FFMQ (Baer et al. 2008; Barnes and Lynn 2010). At high levels of acceptance, i.e., in meditators, awareness (*Observe*) was significantly negatively associated with psychological symptoms (Baer et al. 2008). Therefore, it is possible that a further increase in *Nonreact* could bring a significant negative association between awareness (*Observe*) and sleep problems.

According to the MAT, attention monitoring/awareness was the key for improving cognitive outcomes and enhancing attention to both positive and negative affective information (Lindsay and Creswell 2016). In subjects with sleep difficulties, however, who have a higher tendency to ruminate and bias to perceived internal and external threats to sleep (Zoccola et al. 2009; Harris et al. 2015), high dispositional awareness or cultivation of attention monitoring skills/awareness alone may exacerbate rumination and attentional bias to negative thoughts and generate more psychological distress, resulting in even poorer sleep quality. Therefore, cultivation of both attention monitoring/awareness and acceptance are essential for reducing psychological stress, which are the mechanisms of mindfulness-induced positive influence on stress-related health outcomes (Lindsay and Creswell 2016). Awareness and acceptance together may facilitate the disengagement from ruminated thoughts and daily stress, resulting in reduced psychological stress and arousal, hence shorten sleep latency and improve sleep quality in poor sleepers. Therefore, our findings support the MAT in the context of sleep problems.

Awareness is a monitor of the inner and outer environment, whereas, attention is a process of focusing conscious awareness (Westen 1999). It can be expected that being more attentive to one’s inner psychological stress could result in having more sleep difficulties. In fact, being aware of something is different from being attentive to something, for example, one could be aware of something without being attended to it. However, attention and awareness are often intertwined in reality (Brown and Ryan 2003). Our findings support that it is mindfulness, awareness of the present moment-to-moment experience without judgment (awareness and acceptance), instead of awareness and/or attention, that is beneficial to sleep (Kabat-Zinn 2003).

Our findings are important for a better understanding of the underlying mechanisms of mindfulness intervention on alleviating sleep problems, which paves the way for future studies of the specific role of acceptance on dissociating the adverse impacts of awareness on psychological distress and poor sleep quality.

## Limitations

There are several limitations in the current study. First, the measurement of awareness and acceptance relied on two facets in the FFMQ that have received some criticisms from the literature. The *Observe* facet that captures attention monitoring/awareness has been proposed to exist only in individuals with meditation experience (Baer et al. 2006, 2008). However, some other studies reported that the five factors model including *Observe* facet had an acceptable goodness-of-fit in non-meditator samples (Deng et al. 2011) as well as in mixed samples (including both meditators and non-meditators) (Hou et al. 2014). Furthermore, the *Nonreact* facet that captured the acceptance has been criticized for its low internal consistency and poor association with the global mindfulness score (Deng et al. 2011; Veehof et al. 2011). In our samples, we found a good correlation between *Nonreact* and the global mindfulness score (*r* = 0.603), and the internal consistency of *Nonreact* was also acceptable (Cronbach alpha = 0.74). In addition, all FFMQ facet scores (except the *Observe* facet score), were negatively associated with psychological symptoms, which is consistent with previous literature (Baer et al. 2006, 2008). Typically, the *Observe* facet is not associated with beneficial outcomes until people gain experience in meditation and their awareness associates with an accepting attitude (Baer et al. 2006, 2008). Given that awareness and acceptance are not necessarily correlated in non-meditators, it is appropriate to assess them using the FFMQ and test the moderation effects of acceptance in our samples. Despite the abovementioned potential drawbacks, the FFMQ is still the most widely used self-report questionnaire for accessing mindfulness (Park et al. 2013). Second, the cross-sectional setting limits our interpretation of the findings. Although directional effects have been posited in the moderated mediation analyses, the causality between dispositional mindfulness and sleep quality cannot be determined without a longitudinal design that manipulates one factor with the other factor being constant. Whether high dispositional mindfulness leads to better sleep quality or the other way around remains undetermined in the current study. Future experimental studies that adapt a longitudinal setting are warranted to confirm the causality posited in this study.

## Electronic supplementary material

ESM 1 (DOC 34 kb)

ESM 2 (DOC 35 kb)
