# Supplementary material for: Potential Mechanisms of Mindfulness in Improving Sleep and Distress
Source: Mindfulness (N Y). 2017 Aug 29;9(2):547–55. doi: 10.1007/s12671-017-0796-9 (PMC5866834; doi:10.1007/s12671-017-0796-9)
Supplement: Supplementary file 1 — (DOC 34 kb) [file 12671_2017_796_MOESM1_ESM.doc]

**Supplementary Table 1: Moderating effects of acceptance (*Nonjudge*) on the relationship between awareness (*Observe*) and general psychological distress (N = 364)**

|  |  |  |  | **95% CI** | |
| --- | --- | --- | --- | --- | --- |
| **Variables** | **β** | **SE** | **p-value** | **Lower bound** | **Upper bound** |
| *Nonjudge* | -0.851 | 0.092 | **<0.0001** | -1.033 | -0.670 |
| *Observe* | -0.198 | 0.085 | **0.0209** | -0.365 | -0.030 |
| Interaction | 0.019 | 0.015 | 0.2051 | -0.010 | 0.048 |
| Age | -0.196 | 0.044 | **<0.0001** | -0.284 | -0.109 |
| Gender | 0.733 | 0.855 | 0.3921 | -0.949 | 2.415 |

β = Unstandardized coefficient; CI = Confidence interval; SE = Standard error. The interaction term was generated by multiplying the mean-centered values of *Nonjudge* and *Observe*. The effects of age and gender were controlled.
