# Supplementary material for: Potential Mechanisms of Mindfulness in Improving Sleep and Distress
Source: Mindfulness (N Y). 2017 Aug 29;9(2):547–55. doi: 10.1007/s12671-017-0796-9 (PMC5866834; doi:10.1007/s12671-017-0796-9)
Supplement: Supplementary file 2 — (DOC 35 kb) [file 12671_2017_796_MOESM2_ESM.doc]

**Supplementary Table 2: Moderating effects of acceptance (*Nonjudge*) on the relationship between awareness (*Observe*) and overall sleep quality** (N = 364)

|  |  |  |  | **95% CI** | |
| --- | --- | --- | --- | --- | --- |
| **Variables** | **β** | **SE** | **p-value** | **Lower bound** | **Upper bound** |
| *Nonjudge* | -0.131 | 0.033 | **0.0001** | -0.195 | -0.067 |
| *Observe* | -0.041 | 0.030 | 0.1789 | -0.100 | 0.019 |
| Interaction | 0.005 | 0.005 | 0.3588 | -0.006 | 0.015 |
| Age | -0.017 | 0.016 | 0.2706 | -0.048 | 0.014 |
| Gender | 0.351 | 0.303 | 0.247 | -0.244 | 0.946 |

β = Unstandardized coefficient; CI = Confidence interval; SE = Standard error. The interaction term was generated by multiplying the mean-centered values of *Nonjudge* and *Observe*. The effects of age and gender were controlled.
